# Clustering and combining pattern of metabolic syndrome components in a rural Brazilian adult population

**DOI:** 10.1590/1516-3180.2013.1314326

**Published:** 2013-08-01

**Authors:** Adriano Marçal Pimenta, Mariana Santos Felisbino-Mendes, Gustavo Velasquez-Melendez

**Affiliations:** I PhD. Professor in the Department of Maternal and Child Nursing and Public Health, School of Nursing, Universidade Federal de Minas Gerais (UFMG), Belo Horizonte, Minas Gerais, Brazil.; II MSc. Doctoral Student in the Department of Maternal and Child Nursing and Public Health, School of Nursing, Universidade Federal de Minas Gerais (UFMG), Belo Horizonte, Minas Gerais, Brazil.

**Keywords:** Metabolic syndrome x, Risk factors, Rural population, Cluster analysis, Obesity, abdominal, Síndrome x metabólica, Fatores de risco, População rural, Análise por conglomerados, Obesidade abdominal

## Abstract

**CONTEXT AND OBJECTIVE:**

Metabolic syndrome is characterized by clustering of cardiovascular risk factors such as obesity, dyslipidemia, insulin resistance, hyperinsulinemia, glucose intolerance and arterial hypertension. The aim of this study was to estimate the probability of clustering and the combination pattern of three or more metabolic syndrome components in a rural Brazilian adult population.

**DESIGN AND SETTING:**

This was a cross-sectional study conducted in two rural communities located in the Jequitinhonha Valley, Minas Gerais, Brazil.

**METHODS:**

The sample was composed of 534 adults (both sexes). Waist circumference, blood pressure and demographic, lifestyle and biochemical characteristics were assessed. The prevalences of metabolic syndrome and its components were estimated using the definitions of the National Cholesterol Education Program - Adult Treatment Panel III. A binomial distribution equation was used to evaluate the probability of clustering of metabolic syndrome components. The statistical significance level was set at 5% (P < 0.05).

**RESULTS:**

Metabolic syndrome was more frequent among women (23.3%) than among men (6.5%). Clustering of three or more metabolic syndrome components was greater than expected by chance. The commonest combinations of three metabolic syndrome components were: hypertriglyceridemia + low levels of HDL-c + arterial hypertension and abdominal obesity + low levels of HDL-c + arterial hypertension; and of four metabolic syndrome components: abdominal obesity + hypertriglyceridemia + low levels of HDL-c + arterial hypertension.

**CONCLUSION:**

The population studied presented high prevalence of metabolic syndrome among women and clustering of its components greater than expected by chance, suggesting that the combination pattern was non-random.

## RESUMO

**CONTEXTO E OBJETIVO:**

A síndrome metabólica é caracterizada pela agregação de fatores de risco cardiovasculares como obesidade, dislipidemia, resistência à insulina, hiperinsulinemia, intolerância à glicose e hipertensão arterial. Este estudo objetivou estimar a probabilidade de agregação e o padrão de combinação de três ou mais componentes da síndrome metabólica em população rural adulta brasileira.

**TIPO DE ESTUDO E LOCAL:**

Estudo transversal, conduzido em duas comunidades rurais da região do Vale do Jequitinhonha, Minas Gerais.

**MÉTODOS:**

A amostra foi constituída de 534 adultos, de ambos os sexos, dos quais foram aferidas a circunferência da cintura, a pressão arterial e características demográficas, do estilo de vida e bioquímicas. Prevalências da síndrome metabólica e seus componentes foram estimados usando a definição da National Cholesterol Education Program - Adult Treatment Panel III. A equação da distribuição binomial foi utilizada para avaliar a probabilidade de agregação dos componentes da síndrome metabólica. O nível de significância estatística estabelecido foi 5% (P < 0,05).

**RESULTADOS:**

Síndrome metabólica foi mais frequente em mulheres (23,3%) que homens (6,5%). A agregação de três ou mais componentes da síndrome metabólica foi maior do que esperada ao acaso. Combinações mais comuns para três componentes da síndrome metabólica foram hipertrigliceridemia + baixos níveis de HDL-c + hipertensão arterial, obesidade abdominal + baixos níveis de HDL-c + hipertensão arterial. Para quatro componentes, obesidade abdominal + hipertrigliceridemia + baixos níveis de HDL-c + hipertensão arterial.

**CONCLUSÃO:**

Na população estudada, a prevalência da síndrome metabólica foi alta entre mulheres e houve agregação dos seus componentes acima do esperado que ocorra ao acaso, sugerindo padrão não aleatório de combinação.

## INTRODUCTION

Metabolic syndrome is characterized by clustering of cardiovascular risk factors such as obesity, dyslipidemia, insulin resistance, hyperinsulinemia, glucose intolerance and hypertension.^1^ This syndrome is recognized as an important public health problem worldwide, due to prevalence greater than 20.0% in adult populations living both in urban and in rural areas,^2^^,^^3^^,^^4^^,^^5^^,^^6^^,^^7^ and also to its strong association with cardiovascular diseases and type 2 diabetes, which are both major causes of death worldwide.^1^^,^^8^^-^^9^

Despite this epidemiological context, the management of metabolic syndrome in clinical practice remains controversial,^10^^,^^11^ primarily because of the random clustering of its components. Moreover, metabolic syndrome is diagnosed based upon the presence of three or more components out of a total of five,^12^ which could lead to a plethora of combination patterns.^13^ These various combinations require different interventions and therapeutic approaches, and this is often neglected in clinical practice.^13^ Nonetheless, accurate management of metabolic syndrome, in order to control the current global epidemics of cardiovascular disease and diabetes mellitus, is of fundamental importance.^14^

In Brazil, these issues have been poorly investigated, particularly in rural areas, given that studies have usually focused on the prevalence of metabolic syndrome and associated factors in urban populations. Furthermore, there may be a need for different approaches towards evaluating metabolic syndrome in rural populations, which are also highly affected by this pathological condition^4^^,^^5^^,^^7^ and thus would benefit from interventions such as establishing preventive strategies and adequate treatment.

## OBJECTIVE

The objective of the present study was to estimate the probability of clustering and the combining pattern of three or more metabolic syndrome components in a rural Brazilian adult population.

## METHODS

### Study population and design

A cross-sectional population-based study was conducted between November 2004 and March 2005 in two communities, Virgem das Graças and Caju, in the rural areas of the municipalities of Ponto dos Volantes and Jequitinhonha, respectively. These communities are located in the Jequitinhonha Valley, in the northeast of the state of Minas Gerais, Brazil.

In other projects conducted in these areas, a census performed by our research group in 2001 showed that 1216 individuals were living in these communities. For the present study, 621 of these subjects were excluded due to the following criteria: age less than 18 years (n = 522); emigration (n = 33); death (n = 6); pregnancy (n = 14); diabetes diagnosis (n = 12); polymerase chain reaction values above 10 mg/l [which could indicate acute infection or inflammation] (n = 31);^15^ and physical impossibilities that compromised anthropometric measurements (n = 3). Moreover, 61 individuals were also lost because of their absence at the time of the survey (n = 47) or refusal to participate (n = 14). Finally, data from 534 participants remained available for analysis.

### Ethics committee approval

This study was approved by the Research Ethics Committee of Universidade Federal de Minas Gerais (UFMG), in accordance with National Health Council Resolution 196/96. All of the subjects who took part in the study were informed about the objectives of the research and their rights as participants, and then were asked to sign a consent form.

### Data collection

An interview was conducted by nurses, in which the participants answered a survey questionnaire covering various aspects of their demographic characteristics (sex, age, skin color, marital status and schooling) and lifestyle characteristics (smoking habits and alcohol consumption). At the conclusion of the interview, a clini­cal evaluation was performed on the participants, which included waist circumference and blood pressure measurements, carried out in triplicate by well-trained staff in accordance with standard procedures.^16^

Blood samples were collected from each participant by means of venous puncture following a fasting period of 12 hours. Serum and plasma aliquots were obtained by centrifugation of each sample, and were appropriately treated and stored in vials maintained at 4 °C until arrival at the laboratory for biochemical analysis, in accordance with the recommended technical specifications for avoiding damage to biological material.

Colorimetric enzymatic methods were used to determine glucose, triglyceride and total cholesterol values using a Roche Cobas Mira Plus analyzer (Roche Diagnostics, Switzerland). The high-density lipoprotein cholesterol (HDL-c) concentration was also determined by means of colorimetric enzymatic assay, following precipitation of the low-density lipoprotein cholesterol (LDL-c) and very low-density lipoprotein cholesterol (VLDL-c) fractions, using phosphotungstic acid and magnesium chloride. The LDL-c concentrations were calculated by applying the Friedewald equation,^17^ since there were no triglyceride values > 400 mg/dl: LDL-c = total cholesterol - (HDL-c + triglycerides/5).

Waist circumference was measured to the nearest millimeter, using a non-extendable measuring tape, and this was done exactly halfway between the margin of the lowest rib and the iliac crest, with participants in a standing posi­tion (accurate to 0.1 cm).^16^

Blood pressure was measured by means of an indirect method, using a sphygmomanometer (mercury manometer), in accordance with the Seventh Report of the Joint National Committee on Prevention, Detection, Evaluation and Treatment of High Blood Pressure.^18^ Measurements were made three times in each participant’s right arm with two-minute intervals between measurements, after an initial resting period of at least five minutes.

### Definition of metabolic syndrome

Metabolic syndrome was diagnosed in accordance with the definition of the National Cholesterol Education Program - Adult Treatment Panel III (NCEP-ATP III), which requires the presence of three or more of the following components:^12^

1. Abdominal obesity: waist circumference ³ 102 cm for men and ³ 88 cm for women.
2. Hypertriglyceridemia: triglycerides ³ 150 mg/dl;
3. Low HDL-c: HDL-c < 40 mg/dl for men and < 50 mg/dl for women;
4. Hyperglycemia: fasting blood glucose ³ 100 mg/dl.^14^
5. Arterial hypertension: systolic blood pressure ³ 130 mmHg and/or diastolic blood pressure (DBP) ³ 85 mmHg and/or hypertension treatment.

### Statistical analyses

The study population characteristics were presented in terms of the absolute and relative frequencies of the demographic and lifestyle variables, stratified by sex. These same procedures were used to present the prevalence of metabolic syndrome and its components. Statistical differences were evaluated by means of Pearson’s chi-square test, and the significance level was set at 5% (P < 0.05).

The analyses on clustering of three or more metabolic syndrome components, independently of their cutoff points, were performed based on the quintile distribution of each factor according to sex. Thus, a given component was determined to be present in an individual if he or she had values in the lowest quintile for HDL-c and the highest quintile for the other factors.

The expected degree of clustering of three or more metabolic syndrome components was estimated by calculating the probability of *d* occurrences for *n* factors, where the probability of each occurrence was 0.20 (extreme quintile). Individual probabilities were calculated from the binomial formula presented below.^19^

$$\left(\begin{array}{c} n\\ d \end{array}\right){\left(0.8\right)}^{n-d}{\left(0.2\right)}^{d}$$

Finally, the expected probability was compared with the observed proportion of subjects in the highest quintile for three or more metabolic syndrome components, using the Pearson chi-square test.

In addition, the proportions of three or more metabolic syndrome component combinations were also calculated stratified by sex. Statistical differences were evaluated by means of the Pearson chi-square test, and the significance level was set at 5% (P < 0.05).

All the analyses were performed using the Statistical Package for the Social Sciences (SPSS) software package for Windows, version 15.0 (SPSS Inc., Chicago, IL, United States).

## RESULTS

The study population was composed of 270 men (50.6%) and 264 women (49.4%). The major demographic and lifestyle characteristics of the subjects, according to sex, are shown in Table 1. The age intervals among the population presented homogenous distribution, with a slightly higher proportion of individuals with ages between 18 and 29 years. Most of the subjects lived with a spouse (69.3%) and were of mixed/black color (75.3%). This last characteristic was observed more frequently among men. The proportion of individuals with less than five years of schooling was high (76.3%), as was the proportion of illiterates (34.5%). The prevalences of alcohol consumption and smoking habit were 23.6% and 30.3%, respectively. These habits were also more frequent among men.

**Table 1.** Study population distribution according to demographic and lifestyle characteristics, stratified by sex. Virgem das Graças and Caju, 2004-2005

Variables	Sex				Total	
	Male		Female			
	n	%	n	%	n	%
Age (years)						
18-29	74	27.4	74	28.0	148	27.7
30-39	55	20.4	58	22.0	113	21.2
40-49	45	16.7	42	15.9	87	16.3
50-59	45	16.7	36	13.6	81	15.2
≥ 60	51	18.9	54	20.5	105	19.7
Skin color*^†^						
White	47	17.4	85	32.2	132	24.7
Mixed/black	223	82.6	179	67.8	402	75.3
Marital status						
Living with partner	182	67.4	188	71.2	370	69.3
Living without partner	88	32.6	76	28.8	164	30.7
Schooling (years)						
Illiterate	103	38.1	81	30.7	184	34.5
1-4	112	41.5	111	42.0	223	41.8
5-8	37	13.7	38	14.4	75	14.0
≥ 9	18	6.7	34	12.9	52	9.7
Smoking habits*						
Nonsmoker	93	34.4	192	72.7	285	53.4
Former smoker	63	23.3	24	9.1	87	16.3
Smoker	114	42.2	48	18.2	162	30.3
Alcohol consumption (grams/day)*^‡^						
No consumption	176	65.2	232	87.9	408	76.4
3.1-20	53	19.6	24	9.1	77	14.4
> 20	41	15.2	8	3.0	49	9.2

*P < 0.05 for differences between sexes; ^†^Mixed/black includes all variations of mixed or black; ^‡^Lowest ethanol consumption equals 3.1 grams/day.

The prevalence of metabolic syndrome and its components, according to sex, are shown in Table 2. Metabolic syndrome was diagnosed in 14.9% of the participants, and was four times more frequent among women than among men (P < 0.05). Concerning metabolic syndrome components, 11.6% of the population presented abdominal obesity, 15.2% hypertriglyceridemia, 44.1% low levels of HDL-c, 10.6% hyperglycemia and 59.7% hypertension. Abdominal obesity and low levels of HDL-c were proportionally higher among women (P < 0.05).

**Table 2.** Prevalence of metabolic syndrome and its components according to sex. Virgem das Graças and Caju, 2004-2005

Variables	Sex				Total	
	Male		Female			
	n	%	n	%	n	%
Metabolic syndrome						
Yes	16	6.5	57	23.3	73	14.9
No	230	93.5	188	76.7	418	85.1
Waist circumference*						
< 102 (♂); < 88 (♀)	266	98.5	206	78.0	472	88.4
≥ 102 (♂); ≥ 88 (♀)	4	1.5	58	22.0	62	11.6
Triglycerides (mg/l)						
< 150	217	87.5	208	82.2	425	84.8
≥ 150	31	12.5	45	17.8	76	15.2
HDL-c (mg/dl)*						
< 40 (♂); < 50 (♀)	75	30.2	146	57.7	221	44.1
≥ 40 (♂); ≥ 50 (♀)	173	69.8	107	42.3	280	55.9
Fasting glucose (mg/dl)						
< 100	221	89.5	219	89.4	440	89.4
≥ 100	26	10.5	26	10.6	52	10.6
Hypertension						
No	105	38.9	110	41.7	215	40.3
Yes	165	61.1	154	58.3	319	59.7

*P < 0.05 for differences between sexes; ♂ = men; ♀ = women; HDL-c = high density lipoprotein cholesterol; hypertension is defined as blood pressure ≥ 130/85 mmHg and/or hypertension due to drug treatment.

**Graph 1.**
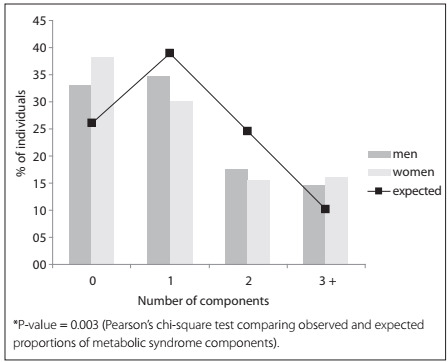
Observed (bar) and expected (line) proportions of individuals for each number of metabolic syndrome components, according to sex. Virgem das Graças and Caju, 2004-2005

Graph 1 presents the observed and expected frequencies of metabolic syndrome components, according to sex. Observed rates are represented by bars while expected rates are represented by lines. The observed frequencies were calculated based on the quintile distribution of metabolic syndrome components and are therefore not identical to the ones shown in Table 2. The expected frequencies were calculated in order to ascertain the nature of the co-occurrence of metabolic syndrome components and were based on the binomial distribution equation. Thus, for both sexes, the clustering of three or more metabolic syndrome components did not occur by chance, since the observed frequency (14.7% for men and 16.0% for women) was higher than the expected frequency (10.2%) (Table 2). Additionally, these proportions were statistically tested and, in fact, this confirmed the differences between the observed and expected proportions (P-value < 0.05).

Table 3 shows the combinations of metabolic syndrome components among the individuals who presented this condition, according to sex. The most common combinations of three components in the population studied were hypertriglyceridemia + low levels of HDL-c + hypertension, and abdominal obesity + low levels of HDL-c + hypertension. The first pattern was more frequent among men and the second, among women. The most frequent combination of four components was abdominal obesity + hypertriglyceridemia + low levels of HDL-c + hypertension. This pattern was also more common among women. On the other hand, the most frequent four-component-pattern presented by men was hypertriglyceridemia + low levels of HDL-c + hyperglycemia + hypertension.

**Table 3.** Combinations of metabolic syndrome components among the participants with this condition, according to sex. Virgem das Graças and Caju, 2004-2005

Combinations	Sex				Total	
	Male		Female			
	n	%	n	%	n	%
Three components						
HTG + LHDL-c + HBP	5	31.3	10	17.5	15	20.5
AO + LHDL-c + HBP	0	0.0	15	26.3	15	20.5
HTG + HGLY + HBP	3	18.8	4	7.0	7	9.6
AO + HGLY + HBP	2	12.5	3	5.3	5	6.8
LHDL-c + HGLY + HBP	3	18.8	1	1.8	4	5.5
AO + HTG + HBP	1	6.3	2	3.5	3	4.1
AO + HTG + LHDL-c	0	0.0	1	1.8	1	1.4
Four components						
AO + HTG + LHDL-c + HBP	1	6.3	8	14.0	9	12.3
AO + LHDL-c + HGLY + HBP	0	0.0	4	7.0	4	5.5
HTG + LHDL-c + HGLY + HBP	1	6.3	3	5.3	4	5.5
AO + HTG + HGLY + HBP	0	0.0	1	1.8	1	1.4
AO + HTG + LHDL-c + HGLY	0	0.0	1	1.8	1	1.4
Five components						
AO + HTG + LHDL-c + HGLY + HBP	0	0.0	4	7.0	4	5.5

HTG = hypertriglyceridemia (triglycerides ≥ 150 mg/dl); LHDL-c = low levels of high density lipoprotein cholesterol (HDL-c < 40 mg/dl for men and < 50 mg/dl for women); HBP = hypertension (blood pressure ≥ 130/85 mmHg and/or hypertension due to drug treatment); HGLY = hyperglycemia; AO = abdominal obesity.

## DISCUSSION

In this study, the prevalence of metabolic syndrome was 14.9%. The degree of clustering of its components was higher than what would be expected by chance and standard combinations between them.

Although the magnitude of the metabolic syndrome was not as high as what was observed in other studies conducted in both urban and rural areas, which was over 20%,^2^^-^^7^ metabolic syndrome is an important public health concern in the study population, since these two communities are located in one of the poorest regions of Brazil. Alternatively, in analyses stratified by sex, the data have demonstrated high prevalence of metabolic syndrome among women (23.3%) and low prevalence among men (6.5%). Similar findings have also been shown in studies developed in rural areas in Brazil^4^ and other countries.^5^^,^^20^ There seems to be a pattern of metabolic syndrome occurrence in rural areas that is characterized by higher prevalence among women. This phenomenon might be determined by people’s occupations in this region, which differ greatly according to sex. Men still perform the field activities that require high energy expenditure, while women are devoted to housework.^5^ Another investigation developed using the same population as in this study showed that men were more active than women in relation to leisure, commuting and work, while women were more active than men in the household domain.^21^

We observed in this study that there were occurrences of three or more metabolic syndrome components at rates greater than would be predicted by chance. This may be indicative of the existence of underlying mechanisms that contribute towards these cluster patterns. This is an important finding, since not all associations among the components have been fully elucidated, thus leading researchers to ask whether the proposed definitions used to diagnose metabolic syndrome might only be random cardiovascular risk factors.^10^^,^^11^ Similar results were found in an investigation conducted on a sample of 4,975 subjects aged between 18 and 74 years from the Framingham Offspring Study, which was an urban population. In that study, it was also pointed out that clustering of three or more cardiovascular risk factors (high levels of total cholesterol, low levels of HDL-c, hypertriglyceridemia, overall obesity, elevated systolic blood pressure and hyperglycemia) occurred at a rate greater than what would be expected by chance and, hence, there ought to be a connection between them.^22^ In other studies conducted on urban populations, it has also been demonstrated that combinations of three or more metabolic syndrome components occurred more frequently than the expected by chance.^23^^,^^24^^,^^25^

Physicians and researchers have been recognizing that cardiovascular disease determinants tend to cluster, and therefore the risk of developing these illnesses rises in line with increases in their clustering abilities.^26^^,^^27^^,^^28^ Therefore, our results corroborate those found in other investigations, thus providing greater consistency regarding the affirmation that metabolic syndrome is clinically useful as a diagnostic tool. Our results also show that, in the rural population studied, the occurrence of clustering of metabolic syndrome components was systematic and not random, thereby reinforcing the hypothesis that underlying pathophysiological mechanisms are involved in this process.

One important criticism of the clinical importance of metabolic syndrome relates to the fact that it is diagnosed based on the presence of three or more components out of a total of five. This creates the possibility of 16 combinations, with different pathophysiological patterns and, consequently, multiple treatment options,^13^ although we only found 13 among the studied population. Additionally, longitudinal studies have demonstrated variations in the risk of mortality according to the different combination patterns of metabolic syndrome components.^29^^,^^30^ In our study, we observed that the most frequent combination of three components was hypertriglyceridemia + low levels of HDL-c + arterial hypertension among men and abdominal obesity + low levels of HDL-c + arterial hypertension among women, while the most common combination of four components was abdominal obesity + hypertriglyceridemia + low levels of HDL-c + arterial hypertension, for the whole study population. These combination patterns are similar to those found in other studies that also used the NCEP-ATP III metabolic syndrome definition.^13^^,^^20^^,^^31^^,^^32^^,^^33^ As a result, it seems that the factors of real relevance in clinical practice are the combination patterns of metabolic syndrome components, according to sex. Consequently, healthcare professionals’ conduct could be guided by these characteristics.

Furthermore, it could be seen that the most frequent combination patterns of metabolic syndrome components in most of the study population were abdominal obesity and dyslipidemia (hypertriglyceridemia and/or low levels of HDL-c). It was only in the pattern of hypertriglyceridemia + low levels of HDL-c + arterial hypertension that co-occurrence of abdominal obesity and dyslipidemia was not observed. On the other hand, this pattern was more frequent among men with an anthropometric profile consisting of low proportions of overall and abdominal obesity, thereby corroborating the findings of other studies.^13^^,^^20^ Obesity, especially the abdominal or visceral type, plays a fundamental role in the pathophysiological mechanism of metabolic syndrome, given that it triggers the insulin resistance pathway as result of excessive free fatty acid accumulation in the blood circulation.^1^^,^^34^

We believe that our findings have a social impact because the study population was still undergoing the process of epidemiological transition, i.e. high rates of morbidity and mortality due to infectious and parasitic diseases^35^ were observed to coexist with increased occurrence of non-communicable illnesses.^36^ Moreover, the local health services are poor, thus hindering the population’s access to actions aimed at health promotion and disease prevention, control and treatment.^35^

Thus, this evidence of clustering and combination patterns of metabolic syndrome components contributes towards the existing knowledge. It corroborates the usefulness of metabolic syndrome as a diagnostic tool with simple clinical-laboratory criteria that are easily applicable at the primary health care level, including isolated rural areas in one of the poorest regions of Brazil.

This study has the following limitations: a) the sample was selected according to convenience, which means that caution is required in interpreting the external validity of our findings; b) the total number of individuals with a positive diagnosis of metabolic syndrome was small (n = 73), which needs to be taken into consideration in evaluating component combination patterns.

## CONCLUSION

The rural population studied here presented high prevalence of metabolic syndrome among women. The metabolic syndrome components presented clustering at a rate greater than what would be expected by chance, suggesting that the combination patterns were non-random. The patterns that were most frequently observed were the following: hypertriglyceridemia + low levels of HDL-c + arterial hypertension; abdominal obesity + low levels of HDL-c + arterial hypertension; and abdominal obesity + hypertriglyceridemia + low levels of HDL-c + arterial hypertension.
